# Automatic Mitochondria Segmentation for EM Data Using a 3D Supervised Convolutional Network

**DOI:** 10.3389/fnana.2018.00092

**Published:** 2018-11-02

**Authors:** Chi Xiao, Xi Chen, Weifu Li, Linlin Li, Lu Wang, Qiwei Xie, Hua Han

**Affiliations:** ^1^Institute of Automation, Chinese Academy of Sciences, Beijing, China; ^2^School of Future Technology, University of Chinese Academy of Sciences, Beijing, China; ^3^Faculty of Mathematics and Statistics, Hubei University, Wuhan, China; ^4^Academy for Advanced Interdisciplinary Studies, Peking University, Beijing, China; ^5^Data Mining Lab, Beijing University of Technology, Beijing, China; ^6^Center for Excellence in Brain Science and Intelligence Technology, Chinese Academy of Sciences, Shanghai, China

**Keywords:** electron microscope, deep learning, volumetric mitochondria segmentation, mitochondria morphology, neuroinformatics

## Abstract

Recent studies have supported the relation between mitochondrial functions and degenerative disorders related to ageing, such as Alzheimer's and Parkinson's diseases. Since these studies have exposed the need for detailed and high-resolution analysis of physical alterations in mitochondria, it is necessary to be able to perform segmentation and 3D reconstruction of mitochondria. However, due to the variety of mitochondrial structures, automated mitochondria segmentation and reconstruction in electron microscopy (EM) images have proven to be a difficult and challenging task. This paper puts forward an effective and automated pipeline based on deep learning to realize mitochondria segmentation in different EM images. The proposed pipeline consists of three parts: (1) utilizing image registration and histogram equalization as image pre-processing steps to maintain the consistency of the dataset; (2) proposing an effective approach for 3D mitochondria segmentation based on a volumetric, residual convolutional and deeply supervised network; and (3) employing a 3D connection method to obtain the relationship of mitochondria and displaying the 3D reconstruction results. To our knowledge, we are the first researchers to utilize a 3D fully residual convolutional network with a deeply supervised strategy to improve the accuracy of mitochondria segmentation. The experimental results on anisotropic and isotropic EM volumes demonstrate the effectiveness of our method, and the Jaccard index of our segmentation (91.8% in anisotropy, 90.0% in isotropy) and F1 score of detection (92.2% in anisotropy, 90.9% in isotropy) suggest that our approach achieved state-of-the-art results. Our fully automated pipeline contributes to the development of neuroscience by providing neurologists with a rapid approach for obtaining rich mitochondria statistics and helping them elucidate the mechanism and function of mitochondria.

## 1. Introduction

Known as the powerhouse of the cell, mitochondria have proven to carry out all types of important cellular functions by producing the overwhelming majority of cellular adenosine triphosphate (ATP). At the same time, they also have substantial responsibility for the regulation of cellular life and death, including disease states. For example, mitochondrial dysfunction has been directly linked to the ageing process, which is the largest risk factor for Alzheimer's disease (AD) (Roychaudhuri et al., 2009). Since morphological alterations usually lead to disturbances in mitochondrial functions and distribution (Mumcuoglu et al., 2012), many meaningful research studies have focused on the relationship between the mitochondrial distribution and shapes and their corresponding functions. Increasing evidence has suggested that the mitochondrial distribution inside a cell can be strikingly heterogeneous (Anesti and Scorrano, 2006). For example, they are often enriched at the cellular sites where the demands for energy are greater or where their metabolic functions are required, such as at the level of the synaptic button. Equally, recent studies have shown that the regulation of mitochondrial shapes is crucial for cellular physiology since changes in mitochondrial shapes have been linked to neurodegeneration, calcium signaling, lifespan, and cell death, which further demonstrates the crucial role that morphological changes in mitochondria play in the immune system (Campello and Scorrano, 2010). Furthermore, it has been established that the function of mitochondria is closely related to cancer (Kroemer, 2006). For example, mitochondria in cancer cells can alter the function of resisting apoptosis (Gogvadze et al., 2008; Wallace, 2012), which has naturally led research studies on cancer therapy to focus on mitochondria by stimulating mitochondrial membrane permeability or by changing the mitochondrial metabolism (Lee et al., 2007). All of these examples show that the statistics and analysis of mitochondria are essential aspects of neurobiological research.

Since mitochondrial structures vary in living cells and the corresponding shapes range from punctuate structures to tubular networks with sizes between 0.5 and 10 μm, optical microscopy, with limited resolution, cannot provide sufficient resolution to reveal these fine structures (Tasel et al., 2016). Fortunately, several new scanning electron microscopy (SEM) imaging methods have emerged, and their high-resolution capability has provided new in-depth insights into mitochondrial structures and functions (Mannella et al., 1997). Each of the available methods involves tradeoffs in terms of resolution, acquisition speed, and reliability. Here, we introduce two representative SEM imaging methods: focused ion beam scanning electron microscopy (FIB-SEM) (Knott et al., 2008) and automated tape-collecting ultramicrotome scanning electron microscopy (ATUM-SEM) (Briggman and Bock, 2012). The FIB-SEM method provides aligned EM images with an isotropic resolution up to 5 × 5 × 5*nm*^3^. However, this method is destructive to tissues since the sections are lost as soon as they are removed from the block face. In contrast, the ATUM-SEM method provides unregistered images with an anisotropic resolution up to 4 × 4 × 30*nm*^3^. It can be applied to large volumes to obtain large-scale statistics and analysis of mitochondrial shapes, and the preserved sections can be imaged and analyzed many times without damaging the tissues. Furthermore, the image acquisition time can be accelerated since the sections collected on the tape can be imaged in parallel using multiple SEMs.

Note that electron microscopy (EM) images with higher resolution will inevitably produce more data from the same volume; thus, mitochondria validation requires a vast amount of laborious manual work. Consequently, an automated mitochondria segmentation method is essential for analyzing large volumes of brain tissue. However, due to the variety of mitochondrial structures, as well as the presence of noise, artifacts and other subcellular structures, automated mitochondria segmentation and reconstruction in EM data have proven to be a difficult and challenging task. In recent years, various attempts have been made to quantify the important properties of mitochondria from EM data. For isotropic image stacks, Jorstad et al. took advantage of the fact that mitochondria have thick dark membranes and proposed an active surface-based method to refine the boundary surfaces of mitochondria for the purpose of segmentation (Jorstad and Fua, 2014). Rigamonti et al. improved on the KernelBoost classifier by iteratively considering the previous segmentation results and original images. The recursion ensured that the classifiers focus on difficult-to-classify locations progressively and exploited the power of the decision-tree paradigm while avoiding over-fitting (Rigamonti et al., 2014). Márquez et al. proposed a non-parametric higher-order model for image segmentation that used a patch-based representation of its potentials (Márquez-Neila et al., 2014). Lucchi et al. put forward an automated mitochondria segmentation method, which utilized an approximate subgradient descent algorithm to minimize the margin-sensitive hinge loss in the structured support vector machine (SSVM) frameworks (Lucchi et al., 2013). For anisotropic image stacks, Tasel et al. first utilized a parabolic arc model to extract membrane structures and then employed the curve energy based on an active contour to obtain the roughly outlined candidate mitochondrial regions (Tasel et al., 2016). Finally, they achieved mitochondria segmentation by means of a validation process in serial section transmission electron microscopy (ssTEM) image stacks (Harris et al., 2006). Márquez et al. presented a computationally efficient approach that worked with anisotropic voxels, allowing the segmentation of large image stacks in serial block-face scanning electron microscopy (SBEM) image stacks (Denk and Horstmann, 2004; Neila et al., 2016). Subsequently, they adopted the conditional random field inference and surface smoothing techniques to improve segmentation and visualization. Perez presented a novel and effective method for segmentation of mitochondria, lysosomes, nuclei and nucleoli in SBEM image stacks. They trained organelle pixel classifiers with the cascaded hierarchical model to generate a probability map and then used active contours to obtain refined results (Perez et al., 2014). In a recent approach, Li et al. used ridge detection to acquire the mitochondrial membrane edges in the variational image segmentation model and further utilized group-similarity in order to optimize the local misleading segmentation (Li et al., 2017a). However, the above methods are traditional machine learning algorithms and rely on hand-crafted features to build classifiers. As the size of the data grows, the performance of these methods will soon reach saturation. Thus, the generalization performance and the segmentation accuracy are not satisfactory enough, and these models are not suitable to handle massive amounts of EM data.

Recently, due to their extraordinary performance, deep neural networks (DNNs) have been widely applied in solving detection and segmentation problems in medical imaging (Ciresan et al., 2012; Beier et al., 2017; Lee et al., 2017). Therefore, the application of DNNs to mitochondria segmentation in EM data holds great promise. Dorkenwald et al. developed the SyConn framework, which also used recursive networks to identify the location of mitochondria (Dorkenwald et al., 2017). However, the proposed networks were mainly focused on synapse segmentation; thus, the accuracy of mitochondria segmentation is not satisfactory. Oztel proposed a fully convolutional network (FCN) to segment mitochondria and then used several types of post-processing, such as 2-D spurious detection filtering, boundary refinement and 3D filtering, to improve the segmentation (Oztel et al., 2017). Xiao et al. proposed an effective approach using a deep network for mitochondria segmentation, which combined Resnet (He et al., 2016) and PSPnet (Zhao et al., 2017) with Inception-net (Szegedy et al., 2015) to deepen the network. However, the above methods applied only 2D FCN to segment mitochondria, which ignored 3D information.

In this work, we proposed a simple yet effective 3D residual FCN for mitochondria segmentation. Inspired by previous studies, we applied a more spatially efficient and better performing architecture to this approach in order to avert the vanishing gradient problem. In the following, we evaluated our approach on both FIB-SEM and ATUM-SEM datasets and compared our results with other promising results obtained by Lucchi et al. (2013), Rigamonti et al. (2014), Ronneberger et al. (2015), Çiçek et al. (2016), He et al. (2016), Li et al. (2017a), Oztel et al. (2017), and Xiao et al. (2018). In summary, the main contributions of this work can be summarized in two different aspects:

- **Method:** We design a fully automated pipeline for mitochondria segmentation and reconstruction based on a 3D convolutional network. This pipeline provides neurologists with a high-efficiency approach for obtaining rich mitochondria statistics. The experimental results on both anisotropy and isotropy datasets suggest that our method achieves state-of-the-art performance.
- **Data:** We provide a public mitochondria database of ATUM-SEM images for facilitating neuroscience research^1^, which consists of the original images and human-labeled ground truth, corresponding to a 17.2 × 16.8 × 1.6 μm^3^ volume.

The remainder of the article is organized as follows. In section 2, we present the datasets and the proposed method in detail. The experimental results and analysis are provided in section 3. Finally, section 4 concludes the paper with some discussions.

## 2. Material and methods

**Figure 1:**
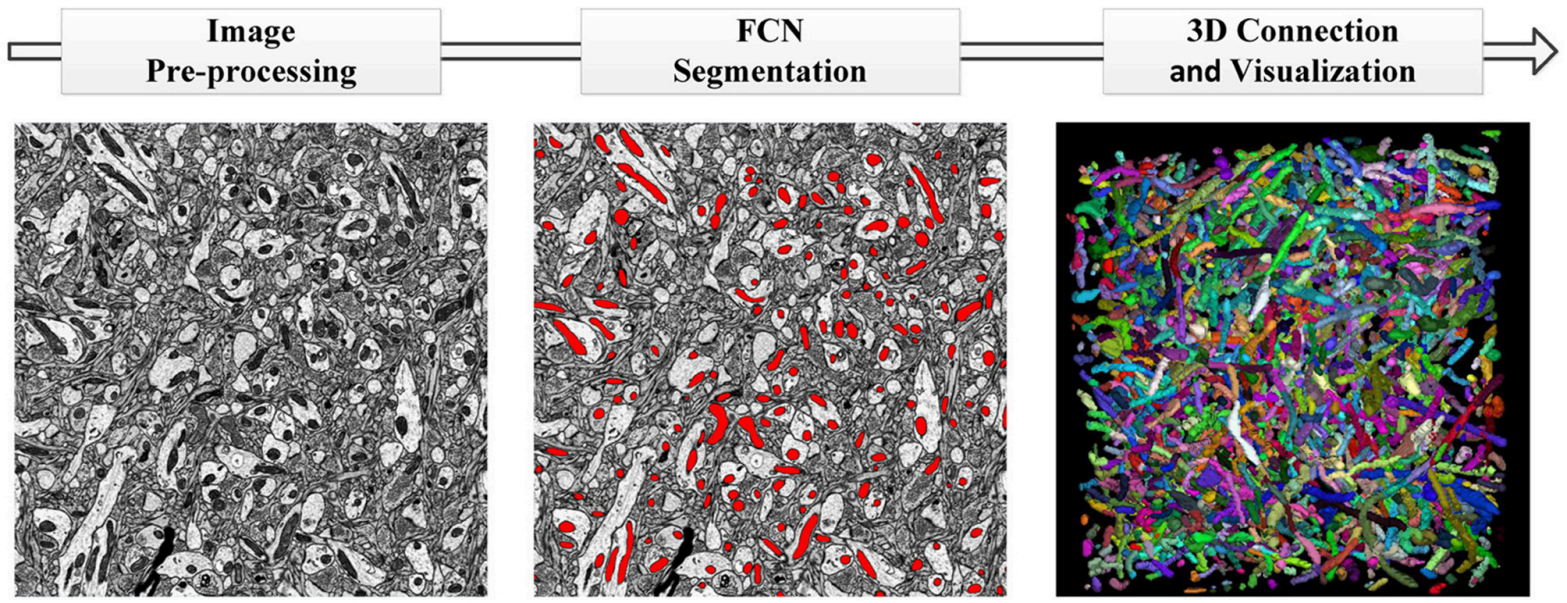
The pipeline of our proposed method. **(Left to Right)** Image pre-processing with registration and histogram equalization; mitochondria segmentation with the proposed 3D residual FCN; 3D connection and visualization in ImageJ.

In this section, we provide a detailed description of the datasets and pipeline of our proposed method. The datasets consist of anisotropic and isotropic EM volumes, which are widely used in the evaluation of mitochondria segmentation. As illustrated in Figure 1, the proposed automated mitochondria segmentation method for EM datasets can be divided into three parts: image pre-processing, mitochondria segmentation with the proposed 3D convolutional network and 3D visualization. All procedures were approved by the Animal Committee of the Institute of Neuroscience, Chinese Academy of Sciences (CAS).

### 2.1. Image datasets

**Figure 2:**
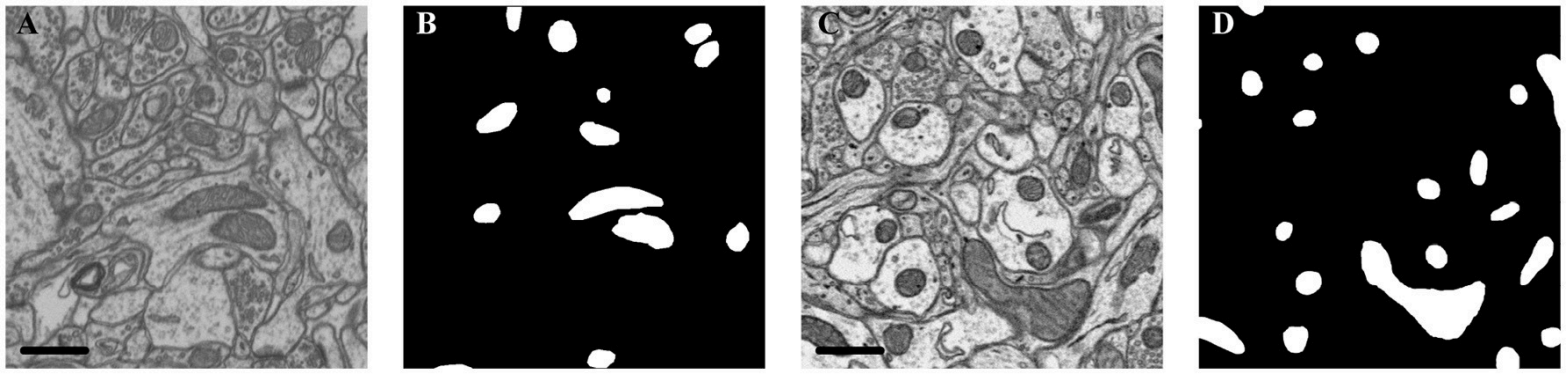
Illustration of the datasets. **(A)** Isotropic image from mouse hippocampus obtained by FIB-SEM; **(B)** Ground truth of the FIB-SEM image; **(C)** Anisotropic image from rat cortex acquired by ATUM-SEM; **(D)** Ground truth of the ATUM-SEM image. Scale bar: 500 nm.

The details of each dataset are summarized in Table 1 and Figure 2. The public FIB-SEM dataset^2^ from a rat hippocampus was acquired by Graham Knott and Marco Cantoni at École Polytechnique Fédérale de Lausanne (EPFL) (Lucchi et al., 2013). In this dataset, the mitochondria were annotated in two volumes: training volume and testing volume. The training dataset consists of a stack of 165 slices from the FIB-SEM dataset, which measures approximately 3.84 × 5.12 × 0.83 μm^3^ with a resolution of 5 × 5 × 5 nm^3^ per voxel. The testing dataset with 165 slices was obtained from a different part of the same specimens.

**Table 1:** Illustration of two datasets.

**Dataset**	**EM**	**Voxel size (*nm*^3^)**	**Train size**	**Test size**
Cortex	ATUM-SEM	2 × 2 × 50	8,624 × 8,416 × 20	8,624 × 8,416 × 11
Hippocampus	FIB-SEM	5 × 5 × 5	1,024 × 768 × 165	1,024 × 768 × 165

In the ATUM-SEM dataset from a mouse cortex, which was acquired by the Institute of Neuroscience, CAS, 31 sections with thicknesses of approximately 50 nm were cut automatically (Yang et al., 2016). Next, these sections were imaged through a Zeiss Supra55 microscope at the Institute of Automation, CAS, where the pixel size was set at 2 nm and the dwell time was set at 2 μs. The ground truths were annotated by neuroanatomists using ImageJ (Schmid et al., 2010) with the TrakEm2 plug-in (Cardona et al., 2012). Note that the production of such a ground truth database required a great amount of human effort, which served to justify that automated segmentation is liable to accelerate neuroscience analyses.

### 2.2. Pre-processing

In this subsection, we presented the pre-processing method consisting of image registration and histogram equalization. The details are as follows.

As mentioned above, the data imaged with the ATUM-SEM method were unregistered. The image registration method adopted (Li et al., 2017b) for serial sections of biological tissue was divided into three parts: (1) searching for correspondences between adjacent sections; (2) displacement calculations for the identified correspondences; and (3) warping the image tiles based on the new position of these correspondences. For correspondence searching, we adopted the SIFT-flow algorithm (Liu et al., 2008) to search for correspondences between adjacent sections by extracting equally distributed grid points from the well-aligned adjacent sections. For the displacement calculation, the positions of the identified correspondences were adjusted throughout all sections by minimizing a target energy function, which consisted of a data term, a small displacement term, and a smoothness term. The data term keeps pairs of correspondences at the same positions in the x-y plane after displacement. The small displacement term constrains the correspondence displacements to minimize image deformation. The smoothness term constrains the displacement of the neighboring correspondences. For image warping, we used the moving least squares (MLS) method (Schaefer et al., 2006) to warp each section with the obtained positions. The deformation results produced by MLS are globally smooth to retain the shape of biological specimens. This image registration method not only reflects the discontinuity around wrinkle areas but also retains the smoothness in other regions, which provides a stable foundation for follow-up works.

In addition, many factors, such as differences in slice thickness, polluted or uneven surfaces and an unstable electron beam, will produce an uneven grayscale between images. It is hard to maintain the acquired images with consistent pixel distributions during the imaging process. Occasionally, some images are much brighter than others, and these differences will inevitably increase the complexity of the images and make segmentation more challenging. To this end, histogram equalization was adopted to weaken the noise and enhance the contrast of raw images, which reduced the complexity of the images and improved the segmentation performance.

### 2.3. The proposed mitochondria segmentation network

In this work, we combined the residual block with variant 3D U-Net to extend and deepen the network. There are three advantages to our proposed architecture: (1) the 3D convolution network is capable of exploiting the 3D spatial information from volumetric EM data, (2) the variant U-Net structure and residual convolution module could better extract features, and (3) injected auxiliary classifier layers could help avoid the problem of vanishing gradients.

**Figure 3:**
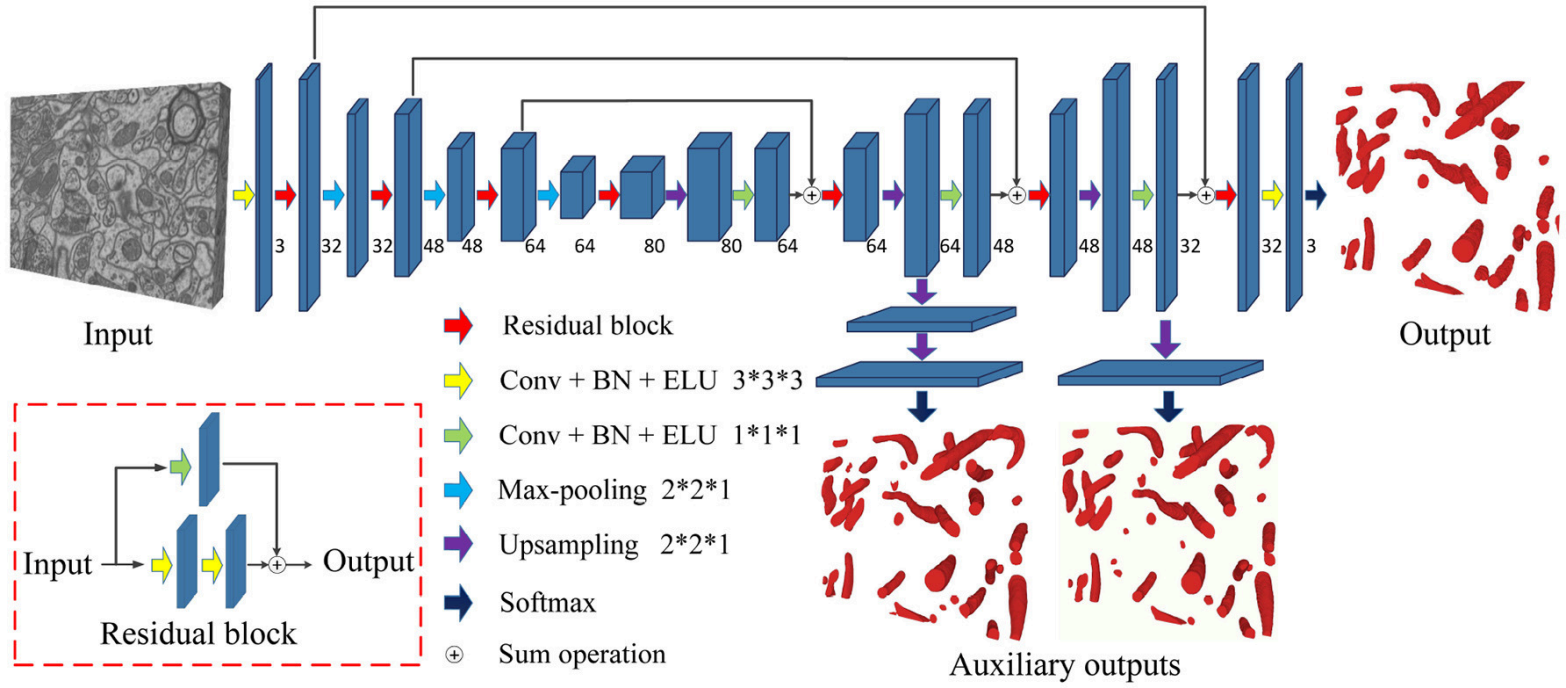
The architecture of the proposed network. Blue blocks denote feature maps, and numbers imply the channel of feature maps in each layer. The red arrow represents the residual block, which consists of two 3D convolutional layers with a kernel size of 3 × 3 × 3 and a residual shortcut connection.

Figure 3 is a pictorial illustration of the proposed network. The components, such as the convolutional layers of the network, are implemented in a 3D manner; hence, the network could effectively extract 3D information from serial EM data. Additionally, the proposed network is a fully convolutional architecture and thus is capable of taking arbitrary-sized 3D data as input and producing corresponding size outputs, which is suitable for dealing with a large-scale serial EM dataset.

As a variant of the U-Net, this network consists of a contracting path and an expansive path. The contracting path contains 13 convolutional layers, and the expansive path contains 15 convolutional layers; thus, the whole network is not entirely symmetric. Specifically, each path could be divided into different stages that operate in different receptive fields, and we adopted a 3D residual module to extract the features in each stage. The 3D residual module consists of two convolutional layers with a kernel size of 3 × 3 × 3 and a residual shortcut connection, each of which was followed by batch normalization (BN) and exponential linear unit (ELU) nonlinearity (to mitigate the internal covariate shift). Zero padding was used to preserve the size of the input feature maps. As confirmed by our empirical experiments, this residual architecture increased the number of ensemble sub-networks and was beneficial for feature extraction. In contrast to previous methods (Milletari et al., 2016; Lee et al., 2017), our residual block contains only two 3D convolutional layers, which maintains the effectiveness and needs fewer parameters.

Given anisotropic ATUM-SEM images with a resolution of 2 × 2 × 50 nm^3^, the widely used max-pooling and upsampling layers (with a stride of 2 × 2 × 2) might lower the resolution of volumes in the z-dimension and reduce the accuracy of predictions. Therefore, we chose the max-pooling and upsampling layers with a stride 2 × 2 × 1 to deal with anisotropy images, which minimized the inferior information loss along the z-dimension.

In the following, several summation-based skip connections were utilized to incorporate global information from higher layers and local cues from lower layers. Compared to concatenation-based skip connections, summation-based skip connections fused multilevel contextual information more thoroughly and helped handle the vanishing gradient problem. To further solve the problem of vanishing gradients, we injected auxiliary classifier layers into the hidden layers to train the network. The details are discussed in section 2.4.

### 2.4. Deeply supervised strategy

Due to the problem of vanishing gradients (Glorot and Bengio, 2010), it is challenging to train such a deep 3D network directly. Motivated by previous studies (Xie and Tu, 2015; Yu et al., 2016; Dou et al., 2017), we utilized a deeply supervised strategy by using injected supervision to train the network. As shown in Figure 3, several upsampling layers (with a stride of 2 × 2 × 1) were inserted into the hidden layers of the network, followed by auxiliary classifier layers. During the training process, the loss of auxiliary classifier layers was added to the total loss of the network with a discount weight (the weight values of the auxiliary losses were 0.15 and 0.3). During the testing process, these auxiliary networks were discarded. This deeply supervised strategy would propagate the back-propagation of the gradient back to the early layers and effectively alleviate the vanishing gradient problem. The total cross-entropy loss function is defined as

(1) $$\begin{array}{l} ℒ\left(X;\theta \right)=ℒ\left(X;W\right)+\sum_{c}[\omega_{c}{ℒ}_{c}(X;W,W_{c})]\\ \;+\frac{\lambda }{2}\left(\sum_{c}{\left\|W_{c}\right\|}_{2}^{2}+{\left\|W\right\|}_{2}^{2}\right). \end{array}$$

In Equation (1), the first two parts are the data loss terms, which include the main and auxiliary classifiers. The last part is the regularization term. Specifically, ω_*c*_ is the weight of the *cth* auxiliary classifier, and λ is used for balancing. $\theta =\left(W,W_{c}\right)$ are the parameters of the proposed 3D fully convolutional network, W denotes the parameters of the network and main classifier, *W*_*c*_ denotes the parameters of the *cth* auxiliary classifier, and X represents the training samples. Therefore, the cross-entropy loss function of the main classifier can be expressed as

(2) $$\begin{array}{l} ℒ\left(X;W\right)=\sum_{i\in Y_{+}}-\log P(y_{i}=1|X;W)\\ \;+\sum_{i\in Y_{-}}-\log P\left(y_{i}=0|X;W\right). \end{array}$$

Here *Y*_+_ and *Y*_−_ represent the mitochondria and non-mitochondria ground truth label sets, respectively. $P\;y_{i}=1|X;W\;\in \left[0,1\right]$ is computed by the softmax function on the activation value at pixel *i*. Similarly, the loss function from the *cth* auxiliary classifier can be expressed as

(3) $$\begin{array}{l} {ℒ}_{c}\left(X;W,W_{c}\right)=\sum_{i\in Y_{+}}-\log P(y_{i}=1|X;W,W_{c})\\ \;+\sum_{i\in Y_{-}}-\log P\left(y_{i}=0|X;W,W_{c}\right). \end{array}$$

### 2.5. Implementation details

#### 2.5.1. Experimental setup

The proposed deep network was implemented using the Keras deep learning library and TensorFlow backend. In the training process, our network was optimized by adaptive moment estimation (Adam) with the following optimization hyperparameters: *learningrate* = 0.0001, exponential decay rates for moment estimates β_1_ = 0.9, β_2_ = 0.999, and *epsilon* = 10^−8^. Binary cross-entropy was chosen as the loss function. It took nearly 77 h to train our network for 30 epochs with a batch size of 2 on a K40 GPU.

#### 2.5.2. Data augmentation

To avoid exceeding the memory of the GPU, smaller images were used to train the proposed network. For the ATUM-SEM dataset, we divided the original ATUM-SEM stack images (size of 8,624 × 8,416 × 20) into numerous small images (size of 256 × 256 × 8). Similarly, we divided the original FIB-SEM stack images (size of 768 × 1,024 × 165) into many small images (size of 256 × 256 × 20). Next, we used rotation and flip strategies to enlarge the training dataset. The transformations for each stack images were combinations of rotations by -90, 0, +90, and 180 degrees and vertical flips over the xy-plane and z-plane. Through data augmentation, the number of images in both training sets was greater than 6,000, which was sufficient for training our network.

#### 2.5.3. Inference

Since the same padding strategy was used in the proposed network, the size of the output patch was the same as that of the input patch. However, this strategy utilized zero padding to match the shapes during the convolution operation, which would influence the segmentation accuracy near the edges of the patch. To resolve this problem, we used overlapping patches and simply blended them together at the time of the test. For the ATUM-SEM dataset, the size of the patch was 1,152 × 1,152 × 8, with a 256 pixel overlap with the xy-plane and 5 pixel overlap with the z-plane. For the FIB-SEM dataset, the size of the patch was 448 × 576 × 20, with a 128 pixel overlap with the xy-plane and 10 pixel overlap with the z-plane.

In addition, due to the effectiveness of test-time augmentation, it has been widely applied in improving the accuracy of segmentation in EM datasets (Quan et al., 2016; Fakhry et al., 2017). In this paper, we applied 16 variations of test-time augmentation to further improve the segmentation results. The testing images were rotated by 90° and flipped over the xy-plane as well as in the z-dimension before passing into the proposed network, and then we applied a reverse transformation to each probability map and took the average of all variations as the final result.

### 2.6. 3D connection method

Because the results produced by the proposed network were probability maps, which were classified into mitochondria and non-mitochondria, it was hard to obtain the shape and statistics of individual mitochondria from these segmentation results.

To display the 3D reconstruction of each mitochondria and obtain the mitochondrial biological statistics, it was necessary to calculate the relationship of mitochondria in 3D. As an approximate solution, the assumption that the 26-connected component of the segmentations belong to the same structure is commonly used. In this paper, we judge the connectivity by calculating the intersection over union (IoU) of two segmentations in adjacent slices. Considering that the small connected components would bias the counting estimations, we discard those components with an IoU smaller than the given threshold *T* = 0.1. The main 3D connection procedure is illustrated in Algorithm 1.

**Algorithm 1:**
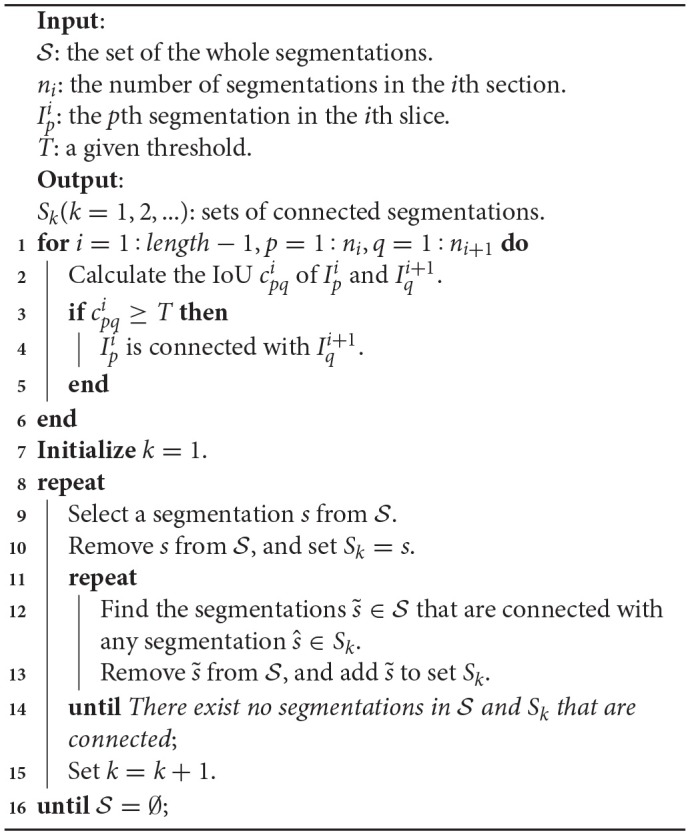
3D Connection Algorithm

With Algorithm 1, we divided the whole segmentations into several disjoint sets, where the segmentations in each set belonged to the same mitochondrion. Next, these segmentation results were imported into ImageJ to display the 3D visualizations.

## 3. Results

In this section, we present the experimental results on the isotropy and anisotropy datasets to demonstrate the effectiveness of the proposed method. We first introduce the evaluation methods. Next, we utilize the different datasets to evaluate the pixel-wise precision of our segmentation method and the accuracy in terms of how many mitochondria are detected correctly in 3D. Finally, we display the 3D reconstruction of mitochondria from large-scale EM datasets and analyse the biological statistics of the mitochondria.

### 3.1. Evaluation methodology

Regarding the evaluation of the pixel-wise segmentation results and detection results, the quantitative results are measured by different metrics. For segmentation evaluation, the results are measured by the Jaccard index, Dice coefficient and conformity coefficient. For detection evaluation, the numbers of true positives (TPs), false positives (FPs) and false negatives (FNs) are computed and used to calculate the precision, recall and F1 score. The details are as follows:

- *Jaccard index*. This metric, which is also known as the VOC score (Everingham et al., 2010), calculates the pixel-wise overlap between the ground truth (Y) and segmentation results (X).
  (4) $$\begin{array}{l} Jaccard\;index\;\left(X,Y\right)=\frac{\text{X}⋂\text{Y}}{\text{X}⋃\text{Y}}. \end{array}$$
- *Dice coefficient*. This metric, which is similar to the Jaccard index, compares the similarity between the ground truth (*Y*) and segmentation results (*X*).
  (5) $$\begin{array}{l} Dice\;coefficient\;\left(X,Y\right)=\frac{2\times |X⋂Y|}{\left|X|+|Y\right|}. \end{array}$$
- *Conformity coefficient*. It is a global similarity coefficient (Chang et al., 2009), which is more sensitive and rigorous than the Jaccard index and Dice coefficient due to its better discrimination capabilities.
  (6) $$\begin{array}{l} Conformity\;coefficient\;\left(X,Y\right)=\frac{2\times Jaccard\left(X,Y\right)-1}{Jaccard\left(X,Y\right)}. \end{array}$$
- *Precision and recall*. These metrics are related to the mitochondria counts in 3D. Precision is the probability that the detected mitochondria are true, and recall is the probability that the true mitochondria are successfully detected.
  (7) $$\begin{array}{l} Precision=\frac{\text{TP}}{\text{TP + FP}}, \end{array}$$
  (8) $$\begin{array}{l} Recall=\frac{\text{TP}}{\text{TP + FN}}. \end{array}$$
- *F*1 *score*. Since precision and recall are often contradictory, this metric is the weighted average of precision and recall, which shows the comprehensive performance of methods.
  (9) $$\begin{array}{l} F1\;score=\frac{2\times \text{Precision}\times \text{Recall}}{\text{Precision + Recall}}. \end{array}$$

Because the shape of mitochondria is sometimes irregular, manual annotations near mitochondria borders are not always accurate, which might influence the precision of the evaluation. Motivated by Li et al. (2017b); Tasel et al. (2016), we defined that a predicted mitochondria is considered a TP only if the voxel-wise overlap between the prediction and corresponding ground truth reaches at least 70%. For the sake of completeness, we also conducted several experiments by considering different voxel-wise overlapping thresholds for TP on both datasets. Details are shown in section 3.3.

### 3.2. Segmentation accuracy

For the sake of completeness, we evaluated the pixel-wise segmentation performance of our approach and compared it with recent mitochondria segmentation methods on both EM datasets. As shown in Tables 2, 3, for the ATUM-SEM dataset, our approach yields 91.8% Jaccard index, 95.7% Dice coefficient and 91.0% conformity coefficient, indicating that it outperforms other methods on all metrics. For the FIB-SEM dataset, the Jaccard index (90.0%) of our approach is higher than those of most algorithms, demonstrating that this approach achieves state-of-the-art results. In addition, the approach with auxiliary classifiers outperforms the method without auxiliary classifiers, and the number of trainable parameters in our proposed 3D network is much smaller than that in other 2D or 3D convolutional networks.

**Table 2:** Mitochondria segmentation performance measured by the Jaccard index, Dice coefficient and conformity coefficient on the ATUM-SEM dataset.

**Methods**	**Jaccard (%)**	**Dice (%)**	**Conformity (%)**	**Trainable parameters**
Li	71.2	75.8	70.5	–
3D U-Net	86.1	92.5	83.8	7.3M
U-Net	87.4	93.8	85.3	7.8M
Fusion-FCN	90.4	94.9	89.3	89.9M
Ours	90.9	95.2	90.0	1.1M
Ours (without auxiliary outputs)	90.7	95.1	89.7	1.1M
Ours (test-time aug8)	91.5	95.5	90.8	1.1M
Ours (test-time aug16)	**91.8**	**95.7**	**91.0**	**1.1M**

**Table 3:** Mitochondria segmentation performance on the FIB-SEM dataset.

**Methods**	**Jaccard (%)**	**Dice (%)**	**Conformity (%)**	**Trainable parameters**
Rigamonti	77.6	–	–	–
Lucchi	86.7	90.1	83.6	–
3D U-Net	87.4	93.2	85.4	7.3M
Oztel	90.7	–	–	–
Ours	89.1	94.2	87.6	1.1M
Ours (without auxiliary outputs)	88.2	93.7	86.4	1.1M
Ours (test-time aug8)	89.8	94.6	88.5	1.1M
Ours (test-time aug16)	90.0	**94.7**	**88.7**	**1.1M**

**Figure 4:**
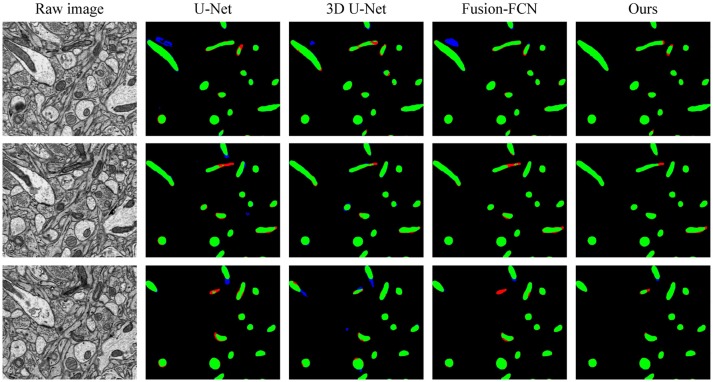
The qualitative comparisons between different methods in three continuous sections from the ATUM-SEM dataset. Green pixels denote TP, red pixels denote FN, blue pixels indicate FP and black pixels denote TN.

**Figure 5:**
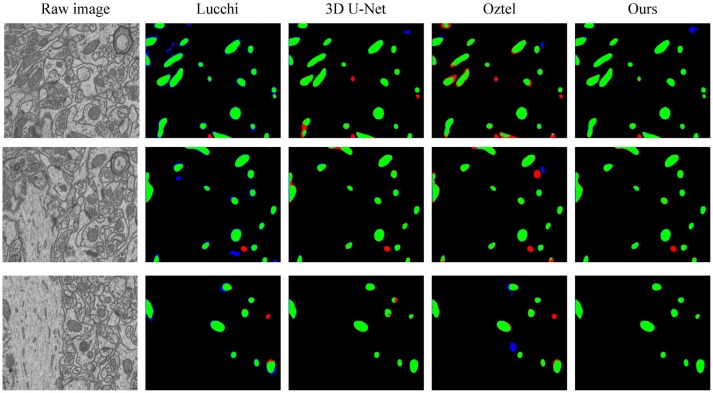
The qualitative comparisons between different methods on the FIB-SEM dataset. Note that our method significantly reduces the number of FNs (red) and FPs (blue).

To explicitly visualize the differences between our results and the results of other methods, we displayed 3 surface-to-surface comparison examples for each dataset in Figures 4, 5, where green pixels denote TP, red pixels denote FN, blue pixels indicate FP and black pixels represent true negative (TN).

Figure 4 shows the segmentation results in three continuous sections from the ATUM-SEM dataset. Owing to the proposed 3D residual convolution network, our approach achieves more accurate results than the methods from Ronneberger et al. (2015), Çiçek et al. (2016), and Xiao et al. (2018). Figure 5 illustrates the segmentation results on the FIB-SEM dataset, where the chosen examples are the same as Oztel et al. (2017). Note that our approach detects most of the mitochondria and effectively reduces FPs as well as FNs, achieving better qualitative results than Lucchi et al. (2013), Çiçek et al. (2016), and Oztel et al. (2017).

### 3.3. Detection accuracy

Until now, we have evaluated our method for pixel-wise segmentation and achieved state-of-the-art results on standard quality metrics. It is also interesting to assess the mitochondria detection performance in 3D. As shown in Figures 4, 5, it might be the case that one algorithm is better at predicting separate mitochondria objects; however, each object is slightly “enlarged” or “shrunken” compared to the ground truth. Is this case worse than one in which the objects are pixel perfect but incorrect mitochondria counts are obtained?

In this case, we evaluated the mitochondria detection performance by the following steps. We first used the connection method shown in 2.6 to perform mitochondria detection in 3D. Next, we removed the detected mitochondria with less than 1,500 voxels, as in Becker et al. (2013). Finally, the numbers of TPs, FPs and FNs are computed and used to calculate the detection accuracy.

**Figure 6:**
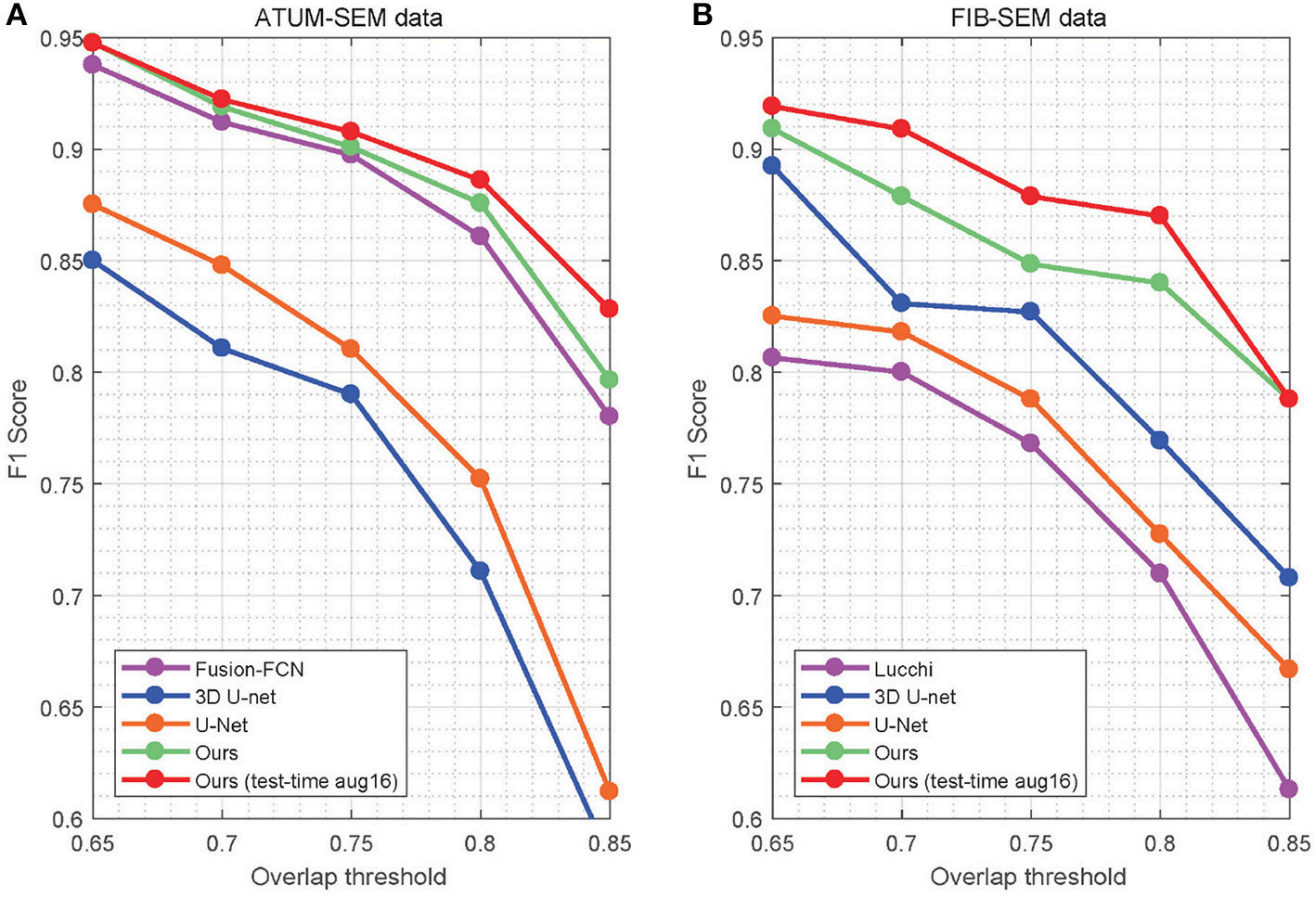
Detection performance of the different overlap thresholds on both EM datasets. Our approach achieves better performance than that of the baseline approaches. **(A)** ATUM-SEM dataset. **(B)** FIB-SEM dataset.

In Table 4, the overlapping threshold for TP is set as 70% and the F1 score of our detection (92.2% in anisotropy, 90.9% in isotropy) outperforms all of the baselines (Lucchi et al., 2013; Ronneberger et al., 2015; Çiçek et al., 2016; Xiao et al., 2018), which suggests that our method achieves state-of-the-art detection performance. Specifically, in the ATUM-SEM dataset, which contains 273 mitochondria in 3D, our approach detects 255 TPs and 25 FPs, and the missed (FN) number is 18. In the FIB-SEM dataset, which contains 32 mitochondria in 3D, our approach obtains 30 TPs and 4 FPs and misses only 2 mitochondria. Additionally, we conducted several experiments by considering different overlapping thresholds for TP on both datasets. As shown in Figure 6, the horizontal axis presents the different overlapping thresholds ranging from 0.65 to 0.85, and the vertical axis denotes the F1 score of the detection results. From Figure 6, it is clear that our method with test-time augmentation yields better performance than other methods for the most of threshold values, which further demonstrates the robustness of our method.

**Table 4:** Quantitative detection performance on two EM datasets.

**EM**	**Methods**	**Precision (%)**	**Recall (%)**	**F1 Score (%)**
ATUM-SEM	U-Net	79.5	90.8	84.8
	3D U-Net	79.1	83.2	81.1
	Fusion-FCN	91.2	91.2	91.2
	Ours	90.4	93.4	91.9
	Ours (test-time aug16)	**91.1**	**93.4**	**92.2**
FIB-SEM	Lucchi	78.8	81.3	80.0
	U-Net	79.4	84.4	81.8
	3D U-Net	81.8	84.4	83.1
	Ours	85.3	90.6	87.9
	Ours (test-time aug16)	**88.2**	**93.8**	**90.9**

Note that our approach yields promising results in both segmentation and detection evaluation, indicating that it is conducive to computing and analysing mitochondria biological statistics such as number, shape and size.

### 3.4. 3D visualization

**Figure 7:**
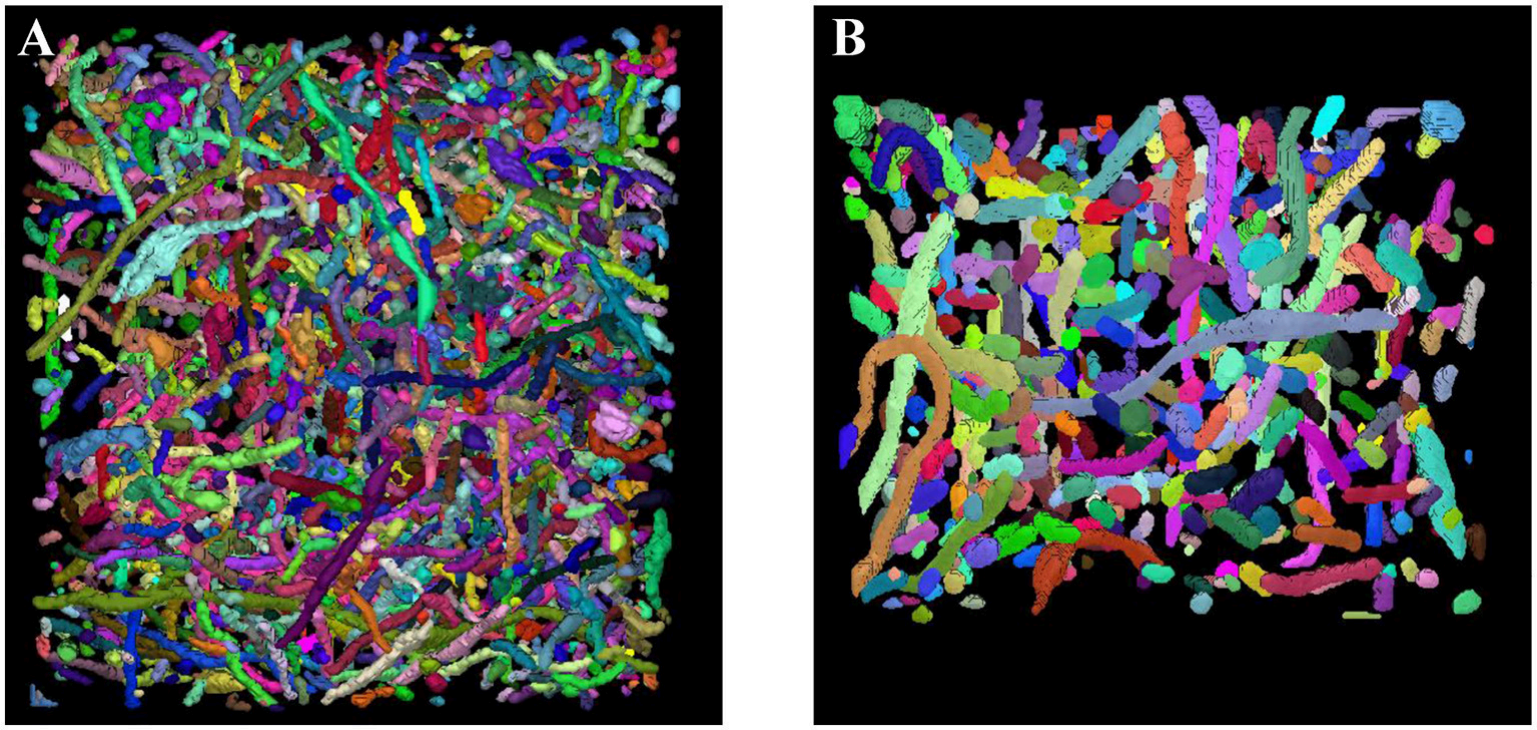
3D reconstruction of mitochondria. **(A)** Reconstruction of mitochondria from the ATUM-SEM dataset with a volume of 15.2 × 17.2 × 8.9 μm^3^; **(B)** Reconstruction of mitochondria from the FIB-SEM dataset with a volume of 10.2 × 7.6 × 5.3 μm^3^.

Sections 3.2 and 3.3 fully demonstrate the effectiveness of our methods. Next, we applied our trained network to the large-scale ATUM-SEM and FIB-SEM datasets, which consist of 15.2 × 17.2 × 8.9 μm^3^ volume and 10.2 × 7.6 × 5.3 μm^3^ volume, respectively. After obtaining the segmentation results, the 3D connection method was utilized to acquire the relationship of mitochondria in 3D. Subsequently, we imported the connection maps into ImageJ, and we display the 3D visualization of mitochondria for each dataset in Figure 7. Here, different colors represent different mitochondria. From the 3D reconstruction results, it can be seen that most mitochondria are intact and continuous, which shows the validity and feasibility of our proposed network and 3D connection method. The corresponding videos are shown in Additional file 1: Video 1 and Additional file 2: Video 2.

### 3.5. Biological statistics

Impairment of mitochondrial morphology by fusion/fission disorder can be detrimental to neuronal cells, resulting in loss of synaptic activity and cell death. Therefore, exploring the 3D morphology of mitochondria is essential for neurological disorder research.

#### 3.5.1. Mitochondrial 3D morphology

**Figure 8:**
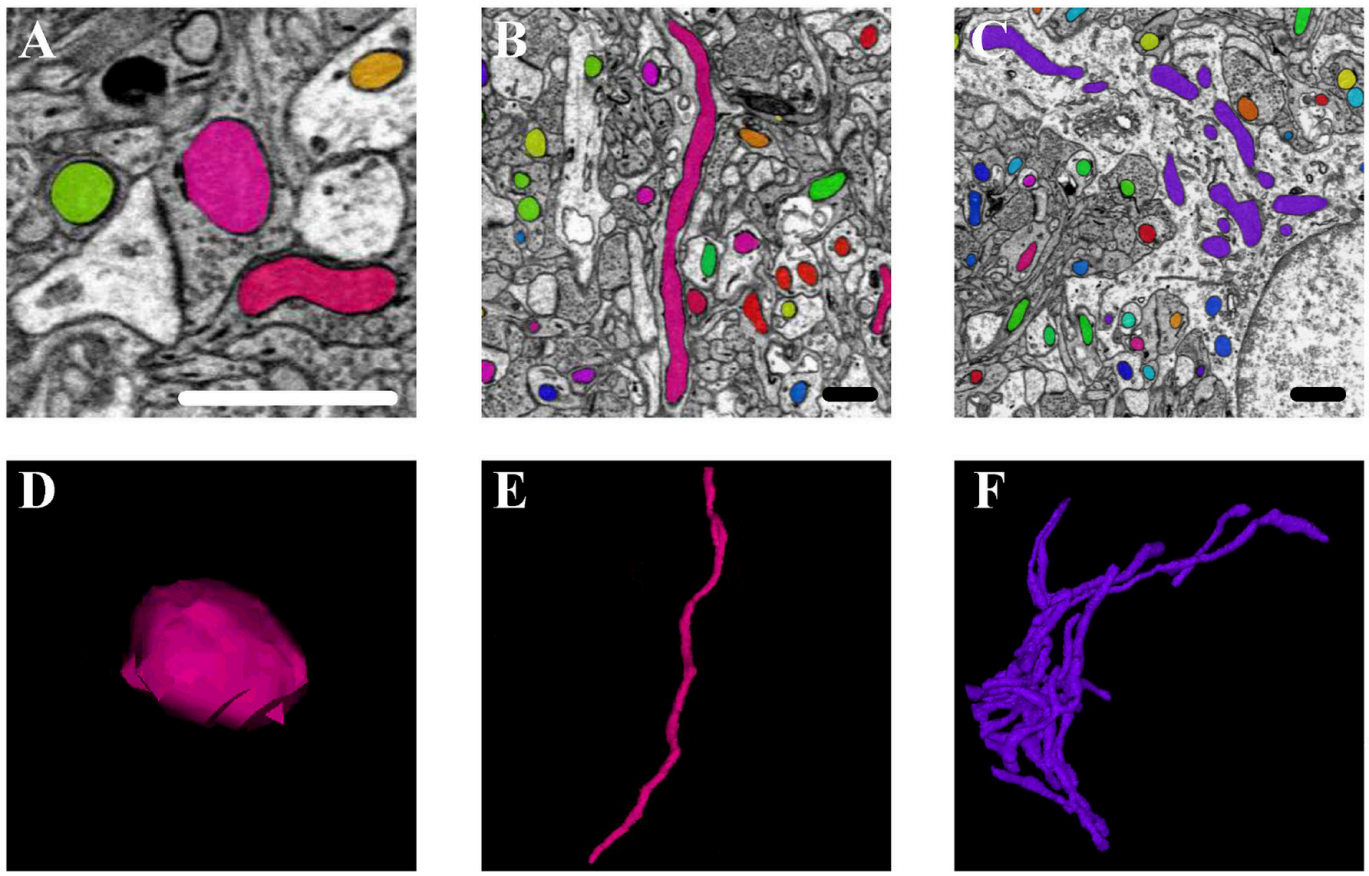
The 3D morphology of mitochondria in different positions in the neurons. **(A,D)** The mitochondria (in pink) positioned at sites of synapse; **(B,E)** the mitochondria (in pink) located in the axons and dendrites; **(C,F)** the mitochondria (in purple) in the cell body. Scale bar: 800 nm.

Mitochondria are highly dynamic organelles that divide and fuse in response to various factors, such as energy requirements, developmental status, and environmental stimulus of the cell. Mitochondria can continuously change their shape through fusion and fission, as well as other processes, such as extension or branching/de-branching. After 3D reconstruction of the mitochondria in the cortex and hippocampus, we found that the mitochondrial morphology in the neurons encompassed a vast spectrum from small spheres and short tubules to elongated tubules and reticular networks depending on their position in the neurons (Figure 8). The mitochondria positioned at sites of synapse, which were proposed to regulate the synaptic activity though energy regulation, were usually flat (Figures 8A,D). In the axons and dendrites, the mitochondrial network was adapted into a long tubular organization that was wrapped around the microtubules. The tubular mitochondria can provide ATP for movement along the axons or dendrites (Figures 8B,E). However, in the cell bodies, the mitochondria typically formed a reticular network radiating from the nucleus. The volume and surface area of the interconnected mitochondria network were approximately 9.05 μm^3^ and 29.72 μm^2^, respectively, which were far greater than those of the mitochondria in the synapse, axons and dendrites (Figures 8C,F). This interconnected mitochondrial system ensures that the neurons are supplied with essential energy and metabolites. These data suggested different morphologies of the mitochondria are associated with the energy-demanding activity of neuron cells.

#### 3.5.2. Measurement of the mitochondrial morphology

**Figure 9:**
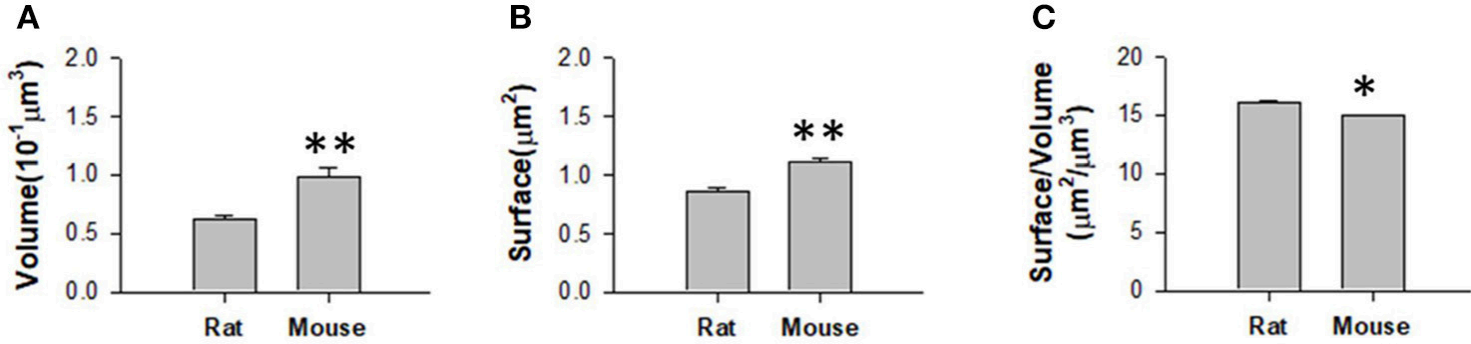
Measurement of the mitochondrial statistics in mouse cortex and rat hippocampus. **(A)** Measurement of the mitochondrial volume in mouse cortex and rat hippocampus. **(B**) Measurement of the mitochondrial surface area in mouse cortex and rat hippocampus. **(C)** Measurement of the mitochondrial volume/surface area ratio in mouse cortex and rat hippocampus. *N* = 319 mitochondria from rat, *N* = 1478 mitochondria from mouse. ^*^P ≤ 0.05,^**^P ≤ 0.01.

We obtained the parameters of the mitochondria morphology in the mouse cortex and rat hippocampus from the 3D reconstruction of mitochondria. As depicted in Table 5, we calculated the mitochondria number, density, volume, surface area, and surface area/volume. The total volume of the mouse cortex (data from ATUM-SEM) was 15.2 × 17.2 × 8.9 μm^3^, including 1,478 mitochondria. The total volume of the rat hippocampus (data from FIB-SEM) was 10.2 × 7.6 × 5.3 μm^3^, including 319 mitochondria. Both the average volume and the average surface area of the mitochondria in the mouse cortex were larger than those of the mitochondria in rat hippocampus, but the surface area/volume ratio was not (Figure 9). Additionally, we quantified the mitochondrial length, width, length/width ratio and flatness in rat and mouse brain samples, and the results are summarized in Table 6. The flatness metric denotes the degree of the flatness, with flat objects having small values close to 0. The above data suggested that the morphology of the mitochondria was associated with the neuron type and the species.

**Table 5:** The statistics of the mitochondria in the large-scale EM datasets.

**Dataset**	**Number**	**Density (N/ *μm*^3^)**	**Average volume**	**Average surface area**	**Surface area/volume**
			**(*μm*^3^)**	**(*μm*^2^)**	
Mouse cortex	1,478	0.6352	0.0928	1.088	14.972
Rat hippocampus	319	0.7764	0.0625	0.855	16.105

**Table 6:** The measurement of mitochondrial morphology in the large-scale EM datasets.

**Dataset**	**Average length**	**Average width**	**Length/width**	**Flatness**
	**(*μm*)**	**(*μm*)**		
Mouse cortex	1.611 ± 1.865	0.412 ± 0.236	3.418 ± 1.842	0.515
Rat hippocampus	1.293 ± 1.132	0.331 ± 0.128	3.597 ± 1.621	0.472

## 4. Discussion

The mitochondrial morphology and networks are regulated via the complex coordination of fission, fusion and distribution events. Defects in the morphology or distribution of mitochondria are correlated with the progression of neurodegenerative diseases such as Alzheimer's, Huntington's and Parkinson's disease. Although much has been learned about mitochondrial morphology in neurological research, the current methods for assessing mitochondrial morphology still leave much to be desired. It is troubling that most current methods for assessing mitochondrial morphology cannot measure mitochondrial morphology objectively. Therefore, we put forward an effective and fast 3D mitochondria segmentation and reconstruction method for large-scale EM data, which provides neurologists with a rapid approach for mitochondrial morphology detection in neuron and neurological disorder research.

As mentioned in Section 3, our approach outperforms several promising approaches on both datasets and achieves state-of-the-art results. For the ATUM-SEM datasets, we compare our results with He et al. (2016), Ronneberger et al. (2015), Çiçek et al. (2016), Li et al. (2017a), and Xiao et al. (2018). By using the 3D convolutional architecture, residual block and deeply supervised strategy, the performance of our approach (Jaccard = 0.918, Dice = 0.957 and Conformity = 0.910) is higher than that of these algorithms. For the FIB-SEM datasets, we compare our results with Lucchi et al. (2013), Rigamonti et al. (2014), Çiçek et al. (2016), and Oztel et al. (2017). As illustrated in Table 3, our method ranks behind only the approach from Oztel et al. (2017), which also utilized a fully convolutional network to segment mitochondria. Note that the above method adopted a series of post-processing methods, which increased the complexity of the algorithm. By comparison, our method achieves satisfactory results without post-processing. Furthermore, Figure 5 illustrates that the qualitative result of our method is better. Although our method yields favorable results for mitochondria segmentation, there is doubt regarding the performance of the proposed method in detection. Thus, we evaluated our method in voxel-wise detection at different overlapping thresholds and compared it with other methods. As shown in Table 4 and Figure 6, the detection results demonstrate the superiority of our method.

The favorable performance of the proposed method can be attributed to the following reasons. First, the use of a 3D fully residual convolution network is beneficial for extracting 3D information and increasing the number of ensemble sub-networks, which helps handle mitochondria segmentation in serial EM data. Another advantage lies in its small number of trainable parameters, which avoids the over-fitting problem. Additionally, we utilize deeply supervised and test-time augmentation strategies to further improve the accuracy of segmentation and detection.

Despite the promising segmentation and detection results, the main limitation of the approach is the high computational cost. For the ATUM-SEM testing dataset (volume of 17.2 × 16.8 × 0.6 μm^3^), the operation times of U-Net, 3D U-Net, Fusion-FCN and our method are 408, 1,776, 3,542, and 2,199 s, respectively. For the FIB-SEM testing dataset (volume of 3.8 × 5.1 × 0.8 μm^3^), the running times of U-Net, 3D U-Net and our method are 66 s, 265 s and 356 s. It can be seen that the proposed method is faster than Fusion-FCN but slower than U-Net and 3D U-Net, which might be ascribed to the complex structure of network. However, the benefit of our method is that it is likely to decrease human proofreading time since its detection and segmentation accuracy is better than that of other approaches. In the future, we will optimize the structure of the proposed network and reduce the computational complexity to improve the speed of the pipeline.

In conclusion, this paper proposes an effective and automated pipeline to segment and reconstruct mitochondria based on deep learning. The experimental results demonstrate the effectiveness of our algorithm in mitochondria segmentation and detection on two different EM datasets. We also implement our pipeline on other larger datasets and obtain measurements of the mitochondrial morphology. The statistics indicate that different morphologies of the mitochondria are associated with the energy-demanding activity of neuron cells, neuron types and species. In addition, we provide a public mitochondria dataset of ATUM-SEM images, which can be used for facilitating neuroscience research.

## Data availability statement

The datasets for this study can be found at http://95.163.198.142/MiRA/mitochondria31 and http://cvlab.epfl.ch/data/em

## Ethics statement

This study was carried out in accordance with the recommendations of the Animal Committee of the Institute of Neuroscience, CAS. The protocol was approved by the Animal Committee of the Institute of Neuroscience, CAS.

## Author contributions

CX conceived and designed the network, developed the algorithm and wrote the paper. XC developed the image registration method. WL implemented the 3D connected algorithm and wrote the paper. HH and QX conceived of the method, gave the research direction and provided feedback on experiments and results. LL and LW were instrumental in analysing the biological statistics.

### Conflict of interest statement

The authors declare that the research was conducted in the absence of any commercial or financial relationships that could be construed as a potential conflict of interest. The reviewer LC declared a shared affiliation, with no collaboration, with one of the authors, LW, to the handling editor at the time of the review.

## Footnotes

^1^http://95.163.198.142/MiRA/mitochondria31/

^2^http://cvlab.epfl.ch/data/em

## Supplementary material

Video 1 3D reconstruction of mitochondria on the ATUM-SEM dataset.

Video 2 3D reconstruction of mitochondria on the FIB-SEM dataset.
